# The Association between Brain-Derived Neurotrophic Factor (BDNF) Protein Level and Body Mass Index

**DOI:** 10.3390/medicina59010099

**Published:** 2022-12-31

**Authors:** Murtada A. Taha, Thekra N. AL-maqati, Yaser A. Alnaam, Sulaiman S. Alharbi, Rajaa Khaneen, Hajra Almutairi, Mashael AL-harbi

**Affiliations:** 1 Clinical Laboratory Sciences Department, Prince Sultan Military College of Health Sciences, P.O. Box 33048, Dammam 31448, Saudi Arabia; 2 Nutrition and Dietetics Department, King Fahad Military Medical Complex, P.O. Box 946, Dhahran 31932, Saudi Arabia; 3 Department of Central Military Laboratory and Blood Bank, Prince Sultan Military Medical City, P.O. Box 7897, Riyadh 11159, Saudi Arabia; 4 Department of Core Lab-Chemistry, Armed Forces Hospital King Abdul-Aziz Naval Base, P.O. Box 413, Jubail 31951, Saudi Arabia

**Keywords:** brain-derived neurotrophic factor (BDNF), body mass index (BMI), obesity, Serum BDNF, adult

## Abstract

*Background and Objectives*: Obesity is a major health concern worldwide. Many studies emphasize the important role of brain-derived neurotrophic factor (BDNF) in regulating appetite and body weight. We aimed to investigate the association between BDNF protein serum levels and body mass index (BMI). *Materials and Methods*: We conducted a cross-sectional study among 108 healthy adult participants divided into six categories depending on their body mass index (BMI). The ages of the participants ranged between 21 to 45 years. The BDNF serum level was measured using the enzyme-linked immunosorbent assay (ELISA) technique. *Results*: A Kruskal–Wallis test showed a significant difference in BDNF between the different BMI categories, χ^2^(2) = 24.201, *p* < 0.001. Our data also showed that BDNF levels were significantly lower in people with obesity classes II and III than those of normal weight (*p* < 0.05). The Spearman rank correlation test was statistically significant with negative correlations between the BMI and BDNF (r) = −0.478, (*p* < 0.01). Moreover, we observed a negative dose-dependent relationship pattern between BMI categories and the levels of circulating BDNF protein. *Conclusions*: In this study, our data support the hypothesis that low serum levels of BDNF are associated with high BMI and obesity in Saudi adults.

## 1. Introduction

Obesity is one of the world’s most serious health issues [1]. The World Health Organization (WHO) defines it as an excessive or abnormal fat accumulation that significantly impacts health. Obesity is identified and classified with the body mass index (BMI), which is commonly used to classify adults as overweight or obese based on weight and height (kg/m^2^) > 30 [2]. Obese people are more susceptible to major diseases and health issues, including cardiovascular disease (CVD), high blood pressure, type II diabetes, and cancers than people with a normal or healthy weight [3,4]. Aside from the health consequences, obesity has an economic impact on and burdens the healthcare system [5,6]. Obesity has become a global epidemic, with at least 2.8 million people dying annually from being overweight or obese [7,8]. Saudi Arabia has one of the highest obesity prevalence rates, with an obese population of 35.2% [9,10]. There is a significant difference between age categories, locations, and both sexes [11]. Obesity in the Saudi population is associated with less physical activity, diet, marital status, hypertension, hypercholesterolemia, diabetes, education, and chronic disease history [12,13,14]. Obesity is considered a multifactorial and complex condition that partly involves an imbalance in energy consumption [15,16]. It is now firmly established that being overweight and many forms of obesity are conditions that have the propensity to concentrate within a family [17]. Many genes are associated with obesity, and the brain-derived neurotrophic factor (BDNF) gene is one of them [18]. The BDNF protein is a 119-amino-acid, 13.6-kDa member of the neurotrophin signaling protein required for the survival and maintenance of peripheral sensory neurons and energy balance [19,20]. In hypothalamic areas, BDNF and its receptor, tropomyosin-related kinase B (TrkB), are mainly produced and assumed to be required for maintaining a healthy weight [21,22]. It would induce a sense of fullness by sending numerous signals to a portion of the hypothalamus in charge of food intake [23]. BDNF is a crucial component of the leptin-signaling cascade involved in energy homeostasis despite little being known about its association with obesity [24]. Several studies suggest that the BDNF gene has a substantial role in regulating eating attitudes, appetite, and feeling of fullness [25].

Alharbi et al. (2014) explored the effect of 11 predominantly appetite-determining SNPs on obesity in the Saudi Arabian population. They provided evidence that rs10767664 and rs3751812 SNPs in the BDNF and fat mass are associated with obesity and obesity-associated (FTO) genes, respectively [26]. The BDNF gene and protein have been linked to obesity in patients with various conditions, including Wilm’s tumor, aniridia, genitourinary abnormalities, and mental retardation contiguous gene syndrome [27]. The BDNF gene and protein have been linked to obesity in patients with various conditions, and a TrKB receptor mutation has been identified in obese children [28]. On the other hand, some studies suggested no association between obesity and low levels of circulating BDNF [29]. Most BDNF studies on obesity have been conducted in animal models with limited human studies [30]. Some animal model studies found that a deleted BDNF gene in mice causes hyperphagic obesity [31]. Furthermore, most human studies have focused on the role of BDNF in obesity among diabetics and those with mental disorders but not on obesity among healthy individuals. Moreover, this type of study has not been previously conducted in Saudi Arabia. For these reasons, we aimed to study the association between serum BDNF levels and BMI among healthy subjects in Saudi Arabia. We hypothesized that lower serum BDNF levels are related to elevated BMI and obesity in Saudi Arabia.

## 2. Materials and Methods

### 2.1. Design and Recruitment

Our cross-sectional study was conducted among 108 adult individuals aged 21–45 to examine the association between body mass index (BMI) and BDNF serum level. The study’s participants were identified and enrolled in collaboration with the Nutrition Department at King Fahad Military Medical Complex (KFMMC) between April 2018 and March 2019. The Nutrition department enrolled subjects and measured their height and weight to calculate BMI depending on our exclusion and inclusion criteria. The participants excluded from the study were those with acute medical conditions, cardiovascular diseases, diabetes mellitus, stress-related disorders, mental disorders, and those using medications affecting body weight. Informed consent was obtained from the participants, and our the Ethics Committee of Prince Sultan Military College of Health Sciences approved our study.

#### 2.1.1. Body Mass Index (BMI) as a Measurement of Obesity

In this study, we used the body mass index (BMI) to classify obese and overweight individuals. BMI is a ratio of an individual weight in kilograms to height in meters squared (kg/m^2^). BMI is calculated using the following formula: BMI (kg/m^2^) = weight/height^2^, (kg/m^2^). BMI was used to divide the participants into six categories according to the classification of the National Heart, Lung and Blood Institute (NHLBI) of the National Institutes of Health (NIH) [32] and the Centers for Disease Control and Prevention (CDC) [33] as follows: Underweight: BMI is <18.5; Normal weight: BMI is 18.5–24.9; Overweight: BMI is 25–29.9; Obesity class I: BMI is 30–34.9; Obesity class II: BMI is 35–39.9, and Extreme obesity class III: BMI is >40 [34].

#### 2.1.2. Serum BDNF Protein Measurement

We used a serum separator tube (SST) to collect 1 mL of the blood samples from 108 participants. After collection, the blood sample was allowed to sit between 30 min and one hour at room temperature to clot before spinning and separating. The blood sample was then centrifuged for 8–10 min at 1500× *g* at room temperature to obtain serum for BDNF measurement. The serum was pipetted, aliquoted into several labeled tubes, and then immediately stored at −80 °C for future use. BDNF was measured using the MOLEQULE-ON ELISA Kit (MOLEQULE-ON, Auckland, New Zealand, Catalog #ELI-M-005-96) according to the manufacturer’s instructions. The samples and 100 μL of the standard were added to an empty 96-well flat-bottom microplate coated with an anti-BDNF monoclonal antibody and incubated for 1 h at room temperature. We ran serum samples and standards in duplicate and diluted 1:2 for this assay. The results were multiplied by a 2-dilution factor. We fitted a 4-parameter logistic curve and determined BDNF concentration in the unknown samples by interpolation from the standard curve. The lower limit of detection for this ELISA assay was 15 pg/mL.

### 2.2. Statistical Analysis

We statistically analyzed our results using SPSS Statistical 26 software. We generated descriptive statistics for sample characteristics and classifications according to BMI. The Kruskal–Wallis test for nonparametric data was used to investigate the differences in BDNF serum levels between the different categories. A post hoc test using the Dunn–Bonferroni approach was conducted. We used Spearman’s rho test to determine the correlations between the BMI and BDNF serum levels. A *p* < 0.05 was considered statistically significant.

## 3. Results

### 3.1. Subject Characteristics

Table 1 shows the demographic characteristics of the study’s 108 subjects. All participants were healthy adults; their ages ranged from 21 to 45; 31% were females, and 69% were males. The participants were divided into six categories based on their body mass index (BMI) ranges according to the NHLBI-NIH and CDC into 15 (14%) underweight, 23 (21%) normal weight, 22 (20%) overweight, while the obesity classes I, II, and III were 19 (18%), 17 (16%), and 12 (11%), respectively.

### 3.2. Relationship of the BDNF Serum Levels with the BMI of the Different Categories

**Figure 1:**
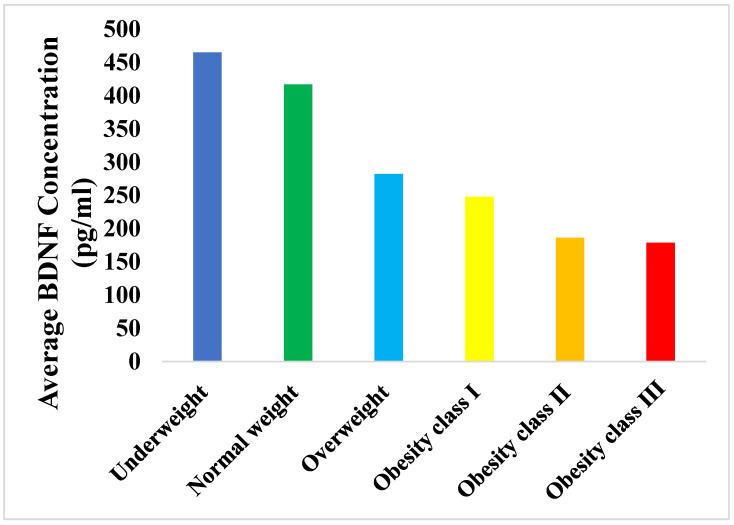
The average serum BDNF (pg/mL) between the different weight categories.

The BDNF concentration means in the serum of the six categories are illustrated in Figure 1. The underweight has the highest concentration with 465.10 pg/mL, followed by normal weight with a mean concentration of BDNF of 417.12 pg/mL. The mean concentration of BDNF in the overweight, obesity class I, II, and III categories were 282.2 pg/mL, 247.9 pg/mL, 186.3 pg/mL, and 178.6 pg/mL, respectively. A Kruskal–Wallis test showed that there was a significant difference in BDNF between the different BMI categories, χ^2^(2) = 24.201, *p* < 0.001 (Table 2). Furthermore, we conducted a post hoc test using the Dunn–Bonferroni approach; the results indicated that BDNF levels were significantly lower in obesity classes II and III than the normal weight (*p* < 0.05). It also showed significant differences between the underweight and obesity classes II and III (*p* < 0.05). However, there were no significant differences between the other categories and normal weight or underweight in terms of BDNF levels (Table 3).

### 3.3. Correlation between BDNF Concentrations and the BMI

We used the Spearman rank correlation to investigate the correlation between BDNF concentrations and the participants’ BMI (kg/m^2^). The BDNF concentration (pg/mL) was statistically significant and negatively correlated with BMI with Spearman rank correlation coefficient (r) = −0.478, (*p* < 0.01) (Table 4).

## 4. Discussion

Obesity is a global health issue. According to researchers, 57.8% of the world’s population will be overweight or obese by 2030 [1,2]. Obesity rates in Saudi Arabia are high between different age categories, locations, and sexes [9,10,11]. Several studies have focused on the role of the BDNF gene and proteins in regulating body weight. However, conflicting results regarding the association between the circulating BDNF protein and obesity measured by BMI have been reported [18,19,20]. Our ELISA test results for BDNF protein in the serum showed that the association between BDNF protein concentrations and BMI as a measure of obesity has a significantly negative correlation (Table 4). A Kruskal–Wallis test showed a significant difference in BDNF between the different BMI categories, χ^2^(2) = 24.201, *p* < 0.001 (Table 2). Furthermore, there were significantly lower BDNF levels in obesity classes II and III than in the normal weight control group (*p* < 0.05). Our results also showed a significant difference between the underweight and obesity classes II and III with *p* < 0.05 (Table 3). According to the WHO, overweight and obese individuals have more fatalities globally than underweight individuals. Obese individuals outnumber underweight people worldwide except in portions of Sub-Saharan Africa and Asia [2,34]. These findings were consistent with Celik Guzel et al. (2014) and Alomari et al. (2020), who found that BDNF levels were significantly lower in obese patients than in the control group [35,36]. El-Gharbawy and colleagues (2006) observed that the serum BDNF protein levels were lower in highly overweight children and adolescents than normal-weight children and adolescents [37]. According to Alharbi et al. (2014) and Lebrun et al. (2006), mutations in the BDNF or TrkB genes are linked to obesity and other eating disorders in humans [38,39]. In experimental animals, BDNF protein deficiency due to variations in the BDNF gene, which causes obesity, is well established [40]. Contrastingly, another study reported no variations in serum BDNF protein concentration levels between two groups of 24 obese and 14 non-obese healthy controls [41]. In another study conducted in Poland involving 144 participants aged 45 to 86, 80 subjects were diagnosed with obesity and 64 with normal body weight. They showed no significant difference in BDNF between the investigated subjects [42].

The contradiction between our results and these studies may be due to factors affecting BDNF measurements, such as smoking, sex, age, food intake, exercise, and ethnicity [25,27]. Moreover, some studies have reported different BDNF concentrations between plasma and serum, where plasma levels are 100 to 200-fold lower than serum [30]. Most of the previous studies compared only two categories, namely obese and normal weight. We used all the BMI categories to reveal for the first time an inverse dose-dependent relationship pattern between the BMI categories and levels of circulating BDNF protein (Figure 1). Our study showed that the underweight category had the highest concentration, with 465.10 pg/mL, followed by the normal-weight control, with a mean BDNF concentration of 417.12 pg/mL, and the overweight and obesity classes I, II, and III, with BDNF protein concentrations of 282.2 pg/mL, 247.9 pg/mL, 186.3 pg/mL, and 178.6 pg/mL, respectively (Figure 1). This interesting finding may have implications and advantages in developing a therapeutic regimen for obesity that suits the different BMI categories. According to Bathina and Das, BDNF influences energy metabolism in rats, resulting in lower calorie intake and body weight reduction through a dose-dependent increase in serotonin turnover [43]. The body relies on cells to store energy, so any changes in genes that regulate this function, of which BDNF is one, can cause an imbalanced bodily state [28,29]. The BDNF gene and protein play several roles in the brain and nervous system; therefore, when they are at higher levels, the feeling of fullness is stimulated by several signals sent to the part of the hypothalamus responsible for food intake [30,31]. As a result, BDNF deficiency leads to the development of obesity because the person would not have any feeling of fullness. Therefore, understanding the factors that contribute to the development of obesity is critical for obesity prevention and treatment measures.

## 5. Conclusions

Obesity has become an epidemic in many societies, causing serious public health and economic problems. It is also associated with increased morbidity, mortality, and a shortened life expectancy. Obesity is considered multifactorial and driven by the complex interactions of environment, genetics, and human behavior. There is some evidence that the BDNF gene and its protein product may be implicated in obesity. However, there are still important gaps in knowledge in this area. In this study, our results showed a significant negative association between BDNF protein concentrations and BMI as a measure of obesity. Furthermore, our results also reveal for the first time an inverse dose-dependent relationship pattern between BMI categories and the levels of circulating BDNF protein.

In summary, the data obtained from this study support our hypothesis that low serum levels of BDNF protein are associated with high BMI and obesity in Saudi adults. This finding may contribute to developing a novel pathophysiological therapeutic tool that enhances the serum level of BDNF protein among obese individuals in Saudi Arabia or worldwide. Future investigations with many subjects are required to understand the mechanism by which BDNF affects obesity, the role of gene mutation in BDNF function, and whether administrating BDNF protein constitutes a new approach to treating obesity.

## Figures and Tables

**Table 1:** Demographic characteristics of the study subjects (*N* = 108).

Age of the Participants (Year; Range)	21–45
Sex	
Female	33 (31%)
Male	75 (69%)

**Table 2:** Kruskal–Wallis test investigating BDNF mean rank between the different BMI categories.

BMI Categories	*N* = 108	Mean Rank BDNF Concentrations	df	X^2^	*p*-Value
Underweight < 18.5	15	70.80			
Normal weight 18.5–24.9	23	73.74			
Overweight 25–29.9	22	53.48	5	24.201	0.0001
Obesity class I 30–34.9	19	48.55			
Obesity class II 35–39.9	17	36.68			
Obesity class III ≥ 40	12	33.79			

**Table 3:** Post hoc analysis of serum BDNF levels between different BMI categories.

Group vs. Group	*p*-Value *
Underweight vs. Obesity class III	0.03
Normal weight vs. Obesity class III	0.005
Underweight vs. Obesity class II	0.03
Normal weight vs. Obesity class II	0.003

* Significance values were adjusted by the Bonferroni correction for multiple tests.

**Table 4:** The correlation between the BDNF concentrations and BMI (Spearman’s rho test).

Variable	BMI (kg/m^2^)
BDNF (pg/mL)	−0.478 **

** Correlation is significant at the 0.01 level (2-tailed).

## Data Availability

Not applicable.
